# Vitamin D and Cancer: An Historical Overview of the Epidemiology and Mechanisms

**DOI:** 10.3390/nu14071448

**Published:** 2022-03-30

**Authors:** Alberto Muñoz, William B. Grant

**Affiliations:** 1 Instituto de Investigaciones Biomédicas “Alberto Sols”, Consejo Superior de Investigaciones Científicas, Universidad Autónoma de Madrid, CIBERONC and IdiPAZ, 28029 Madrid, Spain; amunoz@iib.uam.es; 2 Sunlight, Nutrition and Health Research Center, P.O. Box 641603, San Francisco, CA 94164-1603, USA

**Keywords:** 25-hydroxyvitamin D, 1,25-(OH)_2_D_3_, antitumor action, breast cancer, case-control studies, colorectal cancer, cohort studies, ecological studies, epidemiological studies, randomized controlled trials, UVB, vitamin D

## Abstract

This is a narrative review of the evidence supporting vitamin D’s anticancer actions. The first section reviews the findings from ecological studies of cancer with respect to indices of solar radiation, which found a reduced risk of incidence and mortality for approximately 23 types of cancer. Meta-analyses of observational studies reported the inverse correlations of serum 25-hydroxyvitamin D [25(OH)D] with the incidence of 12 types of cancer. Case-control studies with a 25(OH)D concentration measured near the time of cancer diagnosis are stronger than nested case-control and cohort studies as long follow-up times reduce the correlations due to changes in 25(OH)D with time. There is no evidence that undiagnosed cancer reduces 25(OH)D concentrations unless the cancer is at a very advanced stage. Meta-analyses of cancer incidence with respect to dietary intake have had limited success due to the low amount of vitamin D in most diets. An analysis of 25(OH)D-cancer incidence rates suggests that achieving 80 ng/mL vs. 10 ng/mL would reduce cancer incidence rates by 70 ± 10%. Clinical trials have provided limited support for the UVB-vitamin D-cancer hypothesis due to poor design and execution. In recent decades, many experimental studies in cultured cells and animal models have described a wide range of anticancer effects of vitamin D compounds. This paper will review studies showing the inhibition of tumor cell proliferation, dedifferentiation, and invasion together with the sensitization to proapoptotic agents. Moreover, 1,25-(OH)_2_D_3_ and other vitamin D receptor agonists modulate the biology of several types of stromal cells such as fibroblasts, endothelial and immune cells in a way that interferes the apparition of metastases. In sum, the available mechanistic data support the global protective action of vitamin D against several important types of cancer.

## 1. Introduction

The role of vitamin D in reducing the risk of cancer incidence and death has been studied for years. A search of PubMed on 10 March 2022 searching for “cancer” and “vitamin D” or “vitamin D_3_” in the title or abstract found 6732 publications starting in 1949. Of these, 523 were published prior to 2000; 1630 were published from 2000 through 2009; 1797 were published from 2010 through 2014; and 2782 were published in or after 2015. Publications with vitamin D and cancer in the title or abstract rose from 13 in 1990, 34 in 1995, 75 in 2000, 170 in 2005, 338 in 2010, 401 in 2012, and between 400 and 500 per year since then.

The earliest studies were ecological studies of cancer mortality rates with respect to indices of solar total or UVB radiation or laboratory studies of mechanisms of vitamin D metabolites on cancer cells. As time progressed, observational studies of cancer incidence with respect to serum 25-hydroxyvitamin D [25(OH)D] took place, and studies of the mechanisms of vitamin on cancer incidence, progression, and metastasis were conducted. Later, randomized controlled trials (RCTs) of cancer risk with respect to vitamin D supplementation were conducted, and as more observational studies accrued, meta-analyses were conducted. Along the way, research approaches built on previous studies. However, since there are many sources of vitamin D, UVB exposure, diet, and supplements, and since 25(OH)D concentrations vary with time, both seasonally and over long periods, and since quantifying 25(OH)D concentrations can be uncertain and is not always conducted in studies, all such human studies of vitamin D and cancer are subject to error. There are also methodological issues, such as how to adjust for when 25(OH)D was measured. In addition, what was found in one group of people may not apply to other groups, such as those with different diets, geographical location, clothing, occupation, age, genetics, and BMI. Thus, all the epidemiological studies and RCTs have inherent limitations. However, by taking a comprehensive look at the findings from many types of studies and trying to identify those that are most reliable, a reasonable picture can emerge. What has emerged is that 25(OH)D concentrations play very important roles in the incidence, progression, and death for many types of cancer. While the roles of vitamin D in cancer are not fully understood, there is enough information for clinical and public health decisions to be made.

The epidemiology of vitamin D and cancer can be examined through the prisms of ecological studies, observational studies, and clinical trials. This review looks at findings from ecological studies of cancer risk with respect to indices of solar ultraviolet-B (UVB) doses, observational studies of cancer risk with respect to serum 25(OH)D concentration and oral vitamin D intake, and randomized controlled trials (RCTs) of cancer risk with respect to vitamin D supplementation.

Epidemiological data prompted the study of the putative anticancer action of vitamin D in the laboratory. Two important considerations in the study of the action of 1,25-(OH)_2_D_3_ and analogues in experimental cancer systems are the expression of vitamin D receptor (VDR), which is frequently low or absent, and the high doses of its ligands that are usually required to observe effects. A lack of VDR is linked to transcriptional (by silencing by DNA methylation or repression by SNAIL1/2), posttranscriptional (by several microRNAs) or posttranslational (phosphorylation, alteration of subcellular localization) inhibitory mechanisms, and low cell responsiveness to VDR ligands is often associated with upregulation of the 1,25-(OH)_2_D_3_ degrading enzyme CYP24A1 in tumor cells. These are two reasons for the absence of the 1,25-(OH)_2_D_3_ effects in some studies. An additional consideration is that, though fully convinced of the value of animal models, we will almost exclusively review studies performed in human systems in this paper.

## 2. Epidemiological Studies

### 2.1. Ecological Studies

Ecological studies treat defined populations as entities and compare health outcomes with respect to risk-modifying factors averaged for each population. The groups are usually defined by geographical location but also can be defined by other factors such as occupation. For vitamin D, various indices related to solar UVB dose can be used—for example, annual solar radiation, summertime solar UVB dose, and latitude. Other risk-modifying factors can be added to adjust for confounding factors. Ecological studies offer some advantages: the data required are generally readily available, often with large datasets, and the analyses are easy to do.

Thus, it is not surprising that the first epidemiological study linking vitamin D to a reduced risk of cancer, albeit indirectly, was an ecological study. In 1936, Peller reported that people who developed skin cancer from light exposure, such as from their occupation, had lower rates of internal cancers [1]. In 1937, he showed that sailors in the U.S. Navy, who had extremely high sun exposure, had eight times the expected rate of skin cancer but only 40% of the expected rate of internal cancers [2]. In 1941, Apperly showed that skin cancer mortality rates increased directly in a non-linear fashion with respect to a solar radiation index in the U.S., while total cancer mortality rates decreased in a linear fashion [3]. Evidently, the fact that these three articles were related to vitamin D production went unnoticed until they were cited in a review published in 1993 by Ainsleigh [4].

In 1974, the brothers Cedric and Frank Garland were beginning graduate school at the Johns Hopkins School of Public Health. They attended a lecture by Robert N. Hoover, one author of the *Atlas of Cancer Mortality for U.S. Counties*, *1950–1969* [5]. They were struck by the map for mortality, by county, for cancer of the large intestine except the rectum in white males. It showed low rates in three southwest states and high rates in approximately 15 northeast states. The Garlands reasoned that because vitamin D production is the most important health effect of sun exposure, vitamin D must reduce the risk of cancer in the large intestine (colon). They submitted manuscripts to several journals before one was finally accepted and published in the UK in 1980 [6]. They next found support for their hypothesis in terms of the reduced risk of colorectal cancer with respect to dietary vitamin D and calcium [7], prediagnostic serum 25(OH)D concentration, and risk of colon cancer [8]. They later published early ecological studies on solar radiation and the risk of breast cancer [9] and ovarian cancer [10]. Cedric Garland described their discovery and later work in an online posting at Grassrootshealth.net [11].

In 1999, the National Cancer Institute published the *Atlas of Cancer Mortality in the United States*, *1950–1994* [12]. That revised edition used 10 colors (five shades each of blue and red) to show mortality rates for 38 cancers (see the breast cancer map in Garland’s web post [11] as well as for other cancers at www.sunarc.org, both accessed on 24 February 2022) rather than only five in the earlier version [5]. Data were also displayed for 3053 counties and 506 state economic areas (totals of data for contiguous counties), and showed results for white people (including Hispanics) and black people separately. Through the previous work of one author (W.B.G.) at NASA in Virginia at the time, a map was available of surface-level solar UVB doses in the United States for July 1992 [www.sunarc.org (accessed 24 on February 2022)]. Solar UVB decreases with increasing latitude, albeit with higher doses at any latitude west of the Rocky Mountains than to the east. That effect is due to a combination of higher surface elevation in the west as well as a thinner stratospheric ozone layer owing to the prevailing westerly winds pushing the tropopause up as the air masses cross the Rocky Mountains. Inverse correlations were found for 11 cancers with respect to solar UVB doses for white Americans and several types of cancer for black Americans [13]. A new set of analyses, this time by state, included several risk-modifying factors: alcohol consumption, Hispanic heritage, lung cancer as an index of smoking, poverty status, and urban/rural residence [14]. However, the attribution to solar UVB did not change much between the two articles.

Later, a separate analysis regarding cancer mortality rates for black Americans was published [15]. Significant inverse correlations were found for lung cancer for males and breast cancer for females. The results for colon, esophageal, gastric, and rectal cancer suggested an inverse correlation with respect to solar UVB, but alcohol consumption rates and lung cancer mortality rates also had similar regression coefficients. As a result, UVB did not have a low enough *p*-value to satisfy the Bonferroni criteria. The results were weak because of the lower numbers of black participants in addition to having lower 25(OH)D concentrations [16].

Several ecological studies of UVB and cancer incidence or mortality rates have been published, particularly between 2002 and 2012 [17]. They helped encourage observational studies, mechanism studies, and clinical trials to explore the relationship between vitamin D and cancer. Single-country studies are preferred because people in individual countries tend to have many similarities, such as clothing preferences, diet, and religion, as well as differences, such as smoking, socioeconomic status, and urban/rural residences. Those comparisons can often be modeled. In addition, variations in solar UVB doses tend to be significant [18,19].

Table 1 outlines the more important solar single-country UVB–cancer ecological studies starting in 2002. Most are from mid-latitude countries, but one is from a subtropical country (Iran) and two encompass the Arctic Circle. Most studies used UVB data from NASA’s Total Ozone Mapping Spectrometer (TOMS) satellite instrument [20], but other indices were used as well, including latitude and global solar radiation.

One ecological study was based on data by occupation from a study involving 2.8 million cancer incidence cases from 15 million inhabitants of the five Nordic countries aged 30–64 years in the 10-year censuses from 1960 to 1990 [26]. The study included 53 occupational categories. A novel index, lip cancer less lung cancer, was used for long-term UVB exposure [25]. A suspected important risk factor for lip cancer was solar UVB exposure [27]. A study conducted in Denmark reported that outdoor workers employed for more than 10 years had twice the rate of lip cancer than nonmelanoma skin cancer [28]. Smoking also is a well-known risk factor for lip cancer. As expected, people in occupational categories associated with outdoor work, such as farmers, forestry workers, and gardeners, had the lowest cancer incidence rates.

Table 2 presents findings regarding the incidence of specific cancers for males and females with respect to the UVB indices used. Cancers are listed in descending order of incidence rates in the United States in 2009 to show that as the number of cases decreases, so does the likelihood of finding significant correlations with solar UVB. Note that the results from the United States [22], Russia [24], and the Nordic countries [25] are in good agreement.

Table 3 is similar to Table 2 except for showing mortality rates, not incidence rates, and cancers are listed in descending order with respect to cancer mortality rates in the United States in 2009. Note the good general agreement between the findings for mortality rates in Table 3 with incidence rates in Table 2. The main exception is that solar UVB dose was inversely correlated with mortality rates for several cancers in China, for which it was directly correlated with incidence rates.

### 2.2. Observational Studies Based on Residential UVB Doses

Related to ecological studies of solar UVB and cancer risk are observational studies of ambient solar UVB doses and cancer risk. Cancer incidence data from the prospective National Institutes of Health—AARP Diet and Health Study were used with solar UVB dose data at residential locations to assess the relationship between UVB and cancer risk [30]. The study was limited to participants living in California, Florida, Georgia (Atlanta), Louisiana, Michigan (Detroit), Pennsylvania, and North Carolina. During the 9 years of follow-up, 75,917 participants developed cancer. Erythemal UV data for July from TOMS for 1978–1993 and 1996–2005 were used. Data were adjusted for age; sex; body mass index (BMI); caloric intake; intake of fruit, vegetables, and red and white meat; alcohol consumption; tobacco smoking; education; physical activity; and median household income. Over 9 years of follow-up, UV exposure was inversely associated with total cancer risk (highest vs. lowest quartile) and decreased risk of non-Hodgkin lymphoma and colon, squamous-cell lung, pleural, prostate, kidney, and bladder cancers (all *p*_trend_ < 0.05). UV exposure was associated with increased melanoma risk.

Another example is a nested case–control (NCC) study using 373 esophageal and 249 gastric cancer cases from the UK Biobank with respect to UVB doses at the residential location [31]. Annual solar UVB doses ranged from ~500 kJ/m^2^ in the south to ~750 kJ/m^2^ in the north. Five controls were matched to each case. Data were available for many cancer risk-modifying factors. Significant reductions were found for adjusted esophageal cancer, adjusted lower-third esophageal cancer, and adjusted gastric cancer, in agreement with ecological studies noted previously.

A further discussion of observational studies of cancer incidence and death with respect to solar UVB is in progress.

### 2.3. Observational Studies Based on Serum 25(OH)D Concentrations

Observational studies examine correlations between risk-modifying factors and health outcomes such as cancer incidence, survival, and mortality rates. Observational studies include cohort studies, both prospective and retrospective; case–control (CC) studies; and cross-sectional studies. Each type has advantages and disadvantages. For example, most observational studies regarding vitamin D use serum 25(OH)D concentrations as the index of vitamin D status, but assays used to measure 25(OH)D concentrations vary in quality [32]. Furthermore, serum 25(OH)D concentrations change with the seasons and over long periods [33]. Some studies use dietary vitamin D, i.e., oral vitamin D, including dietary sources and supplements. However, using dietary sources to assess vitamin D intake is problematic because diet generally accounts for less than 300 IU/d in the United States. Although meat is an important source of vitamin D as 25(OH)D [34], most food frequency tables do not include data on meat [35]. Some studies use personal or geographical solar UVB doses. This review emphasizes those that use serum 25(OH)D concentrations but will also include a few that used solar UVB doses.

Generally, CC studies of cancer risk report a stronger reduction with respect to serum 25(OH)D concentrations than do other observational studies. However, observational studies using serum 25(OH)D concentration from blood drawn before cancer diagnosis are generally considered more accurate than those in which blood is drawn near the time of cancer diagnosis.

Researchers have hypothesized that because RCTs have generally not been able to confirm findings from observational studies for many health outcomes, including cancer, having the disease may reduce 25(OH)D concentrations; that is, “reverse causation” [36,37]. However, that effect has been shown only for acute inflammatory diseases such as acute respiratory tract infections [38].

Although systemic inflammation may play a role in cancer risk, the inflammation does not rise as high as in, say, COVID-19. Reports on levels of C-reactive protein levels, an index of systemic inflammation, at the time of diagnosis show that for COVID-19, values can range from 1 to 120 mg/L as severity increases [39], whereas for cancer, they are between 1 and 4 mg/L [40]. Thus, systemic inflammation is not high at the time of cancer diagnosis. We are not aware of any other factor that could result in reverse causality regarding 25(OH)D concentrations for undiagnosed cancer. As will be discussed, the main reason for discrepancies between observational studies and RCTs of vitamin D and cancer is that the RCTs have not been properly designed and conducted.

**Figure 1:**
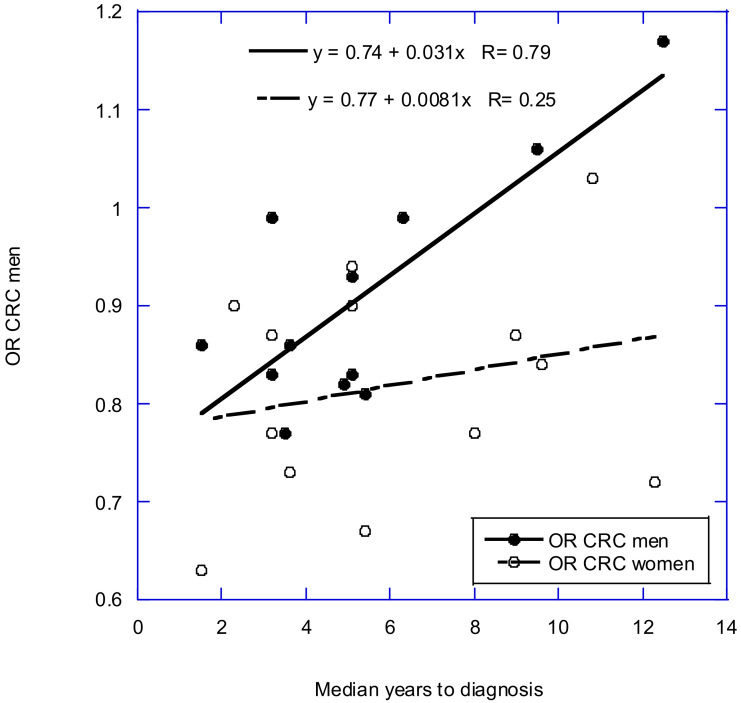
Plot of odds ratio (OR) for CRC against median years to diagnosis for data for men and women used in McCullough and colleagues [47].

Two articles reported that the longer the follow-up time in observational studies of 25(OH)D concentration and cancer risk, the lower the effect of 25(OH)D concentration [41,42]. The same effect has been found for all-cause mortality rates [43]. The reasons include that serum 25(OH)D concentrations change for several reasons and that 25(OH)D concentration near the time of diagnosis is more important than earlier concentrations, even though cancer may develop over a long period. Figure 1 in Grant’s 2012 report [43] shows that the correlation coefficient between serum 25(OH)D concentrations repeated in the same group of participants drops to approximately 0.4 after 14 years.

Most observational studies of 25(OH)D concentration and cancer incidence are prospective cohort or NCC studies. An NCC study of 25(OH)D concentration and incidence of colorectal cancer (CRC) based on two Harvard cohorts [44] is reviewed here to show the complexity of such studies. The Health Professionals Follow-up Study (HPFS), with 18,225 male participants who supplied a blood sample, had 179 cases of CRC during follow-up periods up to 8 years. The analysis of results from the cohort was combined with results from the Nurses’ Health Study (NHS) of women, of whom 32,826 gave blood samples, and 193 developed CRC during 11 years of follow-up [45]. In the HPFS, values for many factors were recorded at baseline in 1994, including season of blood donation, BMI, physical activity, aspirin use, smoking, alcohol intake, intake of vitamin D, calcium and retinol, and meat intake. Analyses were made for colon, rectal, and CRC with respect to quantiles of 25(OH)D, showing that though the trend in 25(OH)D concentrations was not significant for HPFS alone, it was significant when combined with results from NHS. The pooled odds ratio (OR) for CRC for high versus low quintile of 25(OH)D was 0.66 (95% confidence interval [95% CI], 0.42–1.05; *p*_trend_ = 0.01). The risk of rectal cancer increased with respect to 25(OH)D in the HPFS but decreased in the NHS. Interesting findings also were shown for lifestyle characteristics, including BMI, physical activity, calcium intake, retinol intake, and effect of 25(OH)D measured in winter or summer. Thus, with 372 CRC cases, it was possible to find support for 25(OH)D concentrations reducing the risk of colon cancer and CRC.

A meta-analysis published in 2007 based on five NCC studies found a predicted 50 ± 20% reduction in CRC for 34 ng/mL vs. 6 ng/mL [46].

A pooled analysis of 12 NCC studies for CRC for men showed a relative risk (RR) of 0.93 (95% CI, 0.86–1.00), whereas the pooled analysis for 13 studies for women reported an RR of 0.81 (95% CI, 0.75–0.87) [47]. For men and women combined, the RR was 0.87 (95% CI, 0.75–0.87). A significant reduction in RR was shown for women between approximately 25 and 45 ng/mL, but no significant reduction was evident for men at any range. This analysis did not adjust for follow-up time between blood draw and cancer diagnosis. To examine the effect of follow-up time, plots were made of the ORs or RRs from the meta-analysis by McCullough and colleagues [47]. Table 4 shows the data used. Information regarding the relative weight for each study was not available, so plots were made of OR against follow-up time. Figure 1 shows the results. The RR for zero follow-up time should be approximately 0.75 for men and 0.77 for women. The regression fit to the data for men is OR = 0.74 + 0.031x years, *r* = 0.79, adjusted *r*^2^ = 0.59, *p* = 0.002; the regression fit to the data for women is OR = 0.77 + 0.008*x* years, *r* = 0.25, adjusted *r*^2^ = 0, *p* = 0.42. Thus, the lower effect of 25(OH)D on men versus that of women shown in Figure 1 in McCullough and colleagues [47] is due to not accounting for the degradation of the 25(OH)D effect with a longer follow-up time. Providing evidence that the results for men and women should be similar is supported by ecological studies in the United States [14].

In contrast to CRC, prospective and NCC studies with follow-up times greater than 4 years seldom show a significant inverse correlation between serum 25(OH)D concentration and incidence of breast cancer. Breast cancer can develop rapidly, with progression strongly affected by 25(OH)D concentration. Breast cancer is one of the few cancers that have a seasonality in diagnosis, with the highest diagnosis rates in spring and fall [65]. The authors of that study suggested that solar UVB, through producing vitamin D, lowers the risk of breast cancer in summer, whereas higher concentrations of melatonin reduce risk in winter. As a result, many more CC studies of breast cancer with 25(OH)D measured at the time of diagnosis exist than that for CRC.

CC studies of breast cancer incidence with respect to serum 25(OH)D concentrations in pre- and postmenopausal women are discussed first [66,67]. The premenopausal study included 289 cases and 595 matched controls; the postmenopausal study included 1394 cases and 1365 controls. In the premenopausal study, the adjusted OR (aOR) for 25(OH)D >24 ng/mL versus <12 ng/mL was 0.48 (95% CI, 0.29–0.70) and the *p*_trend_ value for the quantiles was 0.0006. In the postmenopausal study, the aOR for 25(OH)D >30 ng/mL versus <12 ng/mL was 0.31 (95% CI, 0.24–0.42) and the *p*_trend_ value of the quintiles was <0.0001. In both studies, the risk increased more rapidly as 25(OH)D concentrations decreased below 12 ng/mL. Those two studies show that several individual factors affect cancer risk but, in general, have little impact on the role of 25(OH)D concentration.

The present study incorporated a search at Google Scholar and the National Library of Medicine’s PubMed database for meta-analyses of cancer incidence or mortality rate with respect to serum 25(OH)D concentration. The most recent meta-analyses were favored. For several cancers, Table 5 includes more than one meta-analysis. Of the 44 studies listed as CC in the meta-analysis of breast cancer by Song and colleagues [68], 26 were true CC studies in which serum 25(OH)D concentration was measured near the time of cancer diagnosis for both cases and controls, with 14,851 cases and 30,979 controls. The remaining 18 studies were NCC studies or, in one case, a cross-sectional study. The number of breast cancer cases was 17,871, whereas the number of controls was 21,753. The analysis for cohort studies of breast cancer incidence in that study included the observational study of breast cancer incidence for participants in either two vitamin D plus calcium RCTs or the Grassrootshealth.net community-based cohort [69]. Because those participants generally had serum 25(OH)D measured every 6 months to 1–2 years, that study should have been combined with the CC studies. It reported an 82% lower risk of breast cancer for 25(OH)D concentration >60 ng/mL versus <20 ng/mL (rate ratio = 0.18 [95% CI, 0.04–0.62]).

From the data in Table 5, it is apparent that CC and NCC studies report greater reductions in cancer risk for high versus low 25(OH)D concentration. The reason may be that cohort studies are conducted for longer than CC or NCC studies. That difference lowers the benefit due to 25(OH)D concentrations as a result of changes in 25(OH)D concentration, as discussed previously. Another finding is that studies of mortality rates show greater reductions than studies of incidence rates. That finding is similar to findings in RCTs of cancer as reported, for example, in the VITAL study [85] as well as in a meta-analysis of results from vitamin D–cancer RCTs [86]. The reason for that finding is probably the presence of many risk-modifying factors that affect cancer incidence but few factors other than vitamin D that affect angiogenesis around tumors, cancer progression, and metastasis into stromal tissue.

Table 6 presents findings from a few meta-analyses of observational studies of vitamin D intake, both from diet and from supplements, and cancer risk. The reductions in cancer risk from oral intake are generally much lower than what is found with respect to serum 25(OH)D concentration studies, largely because differences in oral intakes did not have an observable effect on serum 25(OH)D concentrations. In addition, results with respect to serum 25(OH)D concentrations were not given.

Table 7 presents estimates of the OR for maximum 25(OH)D concentration compared with minimum concentration for several cancers. The reviews obtained from these values did not give numerical values, so they were estimated by inspecting the graphs.

**Figure 2:**
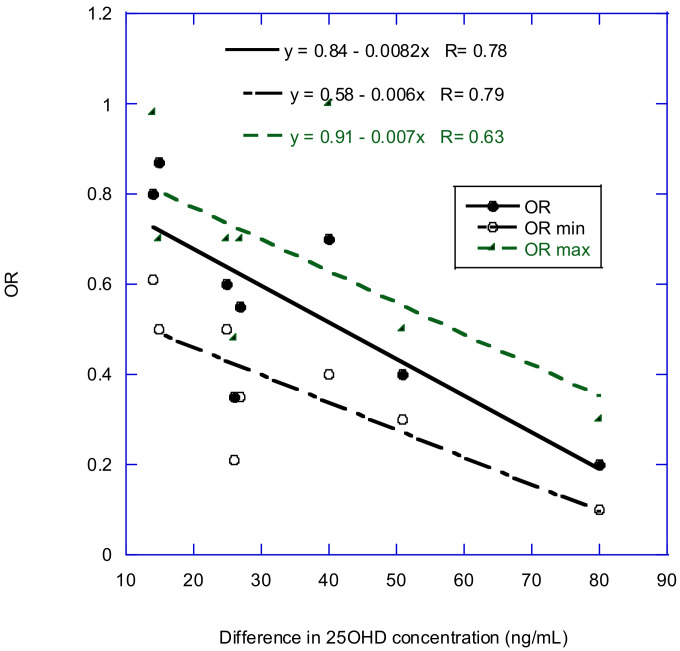
Plot of OR for cancer incidence versus the difference between minimum and maximum 25(OH)D concentration, using data from Table 7, omitting data for all cancer, breast cancer in McDonnell and colleagues [69], and data for prostate cancer.

Figure 2 shows the plot of OR for cancer incidence against the difference between minimum and maximum 25(OH)D concentration. The plot indicates a nearly linear relationship between serum 25(OH)D concentration and OR. The linearity between OR and 25(OH)D concentration is supported by results in the breast cancer study by McDonnell and colleagues [69]. Many studies have few participants with 25(OH)D concentrations above 40 ng/mL, thereby limiting the ability to investigate the effects of higher 25(OH)D concentrations.

### 2.4. RCTs of Vitamin D and Cancer Risk

According to a review published in 2019 [86], nine RCTs have studied how vitamin D supplementation affects cancer incidence, of which five also studied the effect on cancer mortality rate. The relative risk of vitamin D supplementation in the treatment versus placebo groups for cancer incidence was 0.98 (95% CI, 0.93–1.03), whereas for cancer, the mortality rate was 0.87 (95% CI 0.79–0.96). Results did not change significantly if they were analyzed by daily intake versus nondaily intake in a large bolus or attained 25(OH)D concentration >40 ng/mL. However, as pointed out in a recent review by Pilz and colleagues, RCTs rarely found a significant benefit from vitamin D supplementation [91].

The information on most of the trials discussed in [86] plus another published thereafter are presented in Table 8 and Table 9. As can be seen in Table 8, none of the trials were well designed based on what is now known. Not all trials measured baseline 25(OH)D concentration and when they did, the concentrations were almost always above mean population values. Only five reported achieving 25(OH)D concentrations, and both baseline and achieved concentrations were generally based on a fraction of all participants. Four trials used infrequent bolus doses, which were done to improve compliance but resulted in large variations in 25(OH)D concentration between doses since the half-life of 25(OH)D is approximately two weeks. Some of the trials also gave calcium to the treatment arm but not the control arm. In all cases, participants were permitted to take modest vitamin D supplement doses and solar UVB exposure was not controlled. The mean BMI was generally high in the trials, which is a problem since those with higher BMI do not have the same response for a similar change in 25(OH)D concentration as those with lower BMI. For example, the VITAL study [86] reported that participants with BMI <25 kg/m^2^ of body surface area had a significantly reduced risk of cancer from vitamin D supplementation (hazard ratio = 0.76 [95% CI, 0.63–0.90]) but not for higher BMI categories, even though the change in 25(OH)D was near 12 ng/mL for all three BMI categories. The apparent reason is that obesity is an important risk factor for cancer and vitamin D has a limited ability to overcome the mechanisms whereby obesity increases risk of cancer [92]. Finally, only a few of the trials were explicitly designed with cancer incidence a primary outcome.

Only one outcome based on intention to treat was significantly reduced, that of cancer mortality rate in the VITAL trial [85]. Nonetheless, a meta-analysis of five trials found a significant reduction in the cancer mortality rate [86].

The main problem with vitamin D RCTs seems to be that they are generally designed and conducted by following guidelines for pharmaceutical drugs rather than nutrients. For drugs, the only source of the agent is assumed to be what is given to participants in the treatment arm, and a linear dose–response relationship is presumed. Neither assumption is valid for vitamin D. As a result, participants generally have mean 25(OH)D concentrations above the population’s mean values, participants are given small doses of vitamin D, and participants in both the treatment and control arms are permitted to take additional vitamin D supplements as well as produce vitamin D through solar UVB exposure.

Robert Heaney outlined the guidelines for nutrient RCTs in 2014 [101], which were updated in 2018 [102]. The principal guidelines adapted for vitamin D are that:

- Baseline 25(OH)D concentrations should be measured and used as a criterion for inclusion in the study;
- The vitamin D dose should be large enough to increase 25(OH)D concentration to the point at which it would have an observable effect on health outcomes;
- Achieved 25(OH)D concentrations should be measured;
- Conutrient status must be optimized to ensure that vitamin D is the only nutrient-related limiting factor in the response.

No RCT investigating the role of vitamin D in reducing risk of cancer appears to have followed those guidelines.

Some secondary results of the vitamin D–cancer RCTs have yielded useful information. The VITAL study also reported that African American participants had a trend for reduced risk of cancer incidence (hazard ratio = 0.77 [95% CI, 0.59–1.01]). According to the report’s supplementary material for African Americans who supplied 25(OH)D concentration values, the baseline 25(OH)D was 25.0 ng/mL, and the achieved 25(OH)D concentration was 39.7 ng/mL. Those values are in contrast to 31.4 and 42.4 ng/mL, respectively, for non-Hispanic white participants.

In addition, two RCTs showed some effect of vitamin D plus calcium supplementation on risk of cancer [95,98]. When those data were pooled with data from the Grassroots Health volunteer cohort and analyzed by achieved 25(OH)D concentration, the incidence rate of breast cancer for women with 25(OH)D concentrations ≥60 versus <20 ng/mL had a rate ratio of 0.18 (95% CI, 0.04–0.62; *p* = 0.006).

## 3. Perspectives on Epidemiological Studies

### 3.1. Ecological Studies

As would be generally expected, incidence and mortality rates are generally inversely correlated with solar UVB indices unless UVB exposure is linked to increased risk, such as that for melanoma and other skin cancer. The direct correlation with oral cavities and the pharynx in the United States is consistent with UVB exposure’s being a risk factor for lip cancer. Solar UVB exposure increases human papillomavirus (HPV) concentrations, as evidenced by peak rates of positive Pap smears for cervical cancer in Denmark in August [103]. HPV is a risk factor for head and neck cancer [104]. HPV is also hypothesized to be an important risk factor for melanoma [105].

The finding that the incidence rates for several cancers are directly correlated with solar UV in China, whereas most of the cancer mortality rates are inversely correlated, is probably owing to the fact that air pollution levels are much higher in northern than in southern China [106]. In addition, vitamin D generally reduces the risk of cancer mortality rates rather than incidence rates. The reasons may include that although many factors affect cancer incidence, few factors affect cancer progression and metastasis.

Because the countries included are different in many respects, including diet, ethnicity, latitude, and pollution level, ecological studies offer strong evidence that UVB irradiance affects cancers similarly regardless of many other factors.

An important reason why ecological studies have shown robust relationships between indices of solar UVB doses is that they included many cases of cancer. Researchers conducting earlier ecological studies were more likely than researchers of more recent studies to find significant correlations with UVB doses because people back then spent more time in the sun without concern for skin cancer or photoaging, and obesity rates were lower.

### 3.2. Observational Studies

Several findings are important from the analyses presented regarding observational studies.

First, the inverse relationships between serum 25(OH)D concentration and cancer incidence or mortality rates are similar to those between solar UVB and cancer reported in ecological studies. The primary exception is for head and neck cancer; serum risk was inversely correlated with both serum 25(OH)D concentration and vitamin D intake. However, ecological studies showed direct correlations between solar UVB and both incidence and mortality rates for oral cavity/pharynx and pharynx cancers, although one study reported an inverse relationship for laryngeal cancer [25].

Secondly, a long follow-up time was again found to significantly decrease the observed beneficial effect of 25(OH)D concentration. For example, the meta-analysis of CRC risk with respect to 25(OH)D concentration by Hernandez-Alonso and colleagues [72] had 11 studies (one CC, nine NCC, and one meta-analysis) and six prospective cohort studies. The OR for the CC study was 0.45 (95% CI, 0.36–0.57). For the nine NCC studies, the mean follow-up time was near 8 years, and the OR was 0.63, whereas for the prospective cohort studies, the mean follow-up time was 13 years, and the OR was 0.80 (95% CI, 0.66–0.97).

Some parties have argued that CC studies with 25(OH)D concentration measured near the time of diagnosis would be the best type of observational study due to possible reverse causality [53]. There is no evidence to indicate that having undiagnosed cancer reduces 25(OH)D concentration other than perhaps decreasing with the progression cancer stage. Thus, CC studies, which are easier to conduct than prospective studies, are preferred.

The epidemiological and mechanical evidence regarding solar UVB exposure and vitamin D presented here generally satisfy Hill’s criteria for causality in a biological system (based on Kosh’s postulates) [107,108,109]. The only weakness is that RCTs have not yielded strong support, largely because they were poorly designed and conducted. However, as argued by Dr. Thomas R. Frieden, former head of the U.S. Centers for Disease Control and Prevention, in a review in *The New England Journal of Medicine*, RCTs have substantial limitations [110]. The review tabulates the strength and limitations of 11 study designs, including RCTs, prospective cohort, retrospective cohort, case-control, and ecological studies. It concludes by stating that there is no single, best approach to the study of health interventions, and clinical and public health decisions are almost always made with imperfect data.

### 3.3. Historical Overview

Many of the articles reviewed regarding epidemiological studies of solar UVB dose or exposure and vitamin D played important roles in developing the understanding of the role of vitamin D in reducing risk of cancer incidence and mortality rates. Table 10 lists a few of them in chronological order. Note that the importance of some of the articles, notably those reported prior to 1980, was not recognized until many years later.

## 4. Mechanisms Introduction

The first experimental studies supporting this effect of 1,25-(OH)_2_D_3_ were reported in 1981. They addressed the inhibition of human melanoma cell proliferation and the induction of the differentiation of mouse myeloid leukemia cells and were by D. Feldman’s and T. Suda’s groups, respectively [112,113]. Since then, many laboratories have described a high number of antitumoral effects of 1,25-(OH)_2_D_3_ on a variety of molecular mechanisms and cellular processes during carcinogenesis. Previous reviews have discussed some of these mechanisms in particular cancer types [114,115,116,117,118,119]. In this review, we update the current knowledge on 1,25-(OH)_2_D_3_ antitumor mechanisms.

### 4.1. Inhibition of Tumor Cell Proliferation

1,25-(OH)_2_D_3_ exerts an antiproliferative action on tumor cells by direct and indirect mechanisms that are partially redundant and sometimes function simultaneously in target cells. Of note, this action is mostly independent of *TP53* tumor suppressor gene status.

Direct mechanisms. In many cancer cell types, 1,25-(OH)_2_D_3_ directly arrests the cell cycle in the G_0_/G_1_ phase by downregulating cyclin-dependent kinases (CDKs: CDK4, CDK6) and repressing the genes that encode cyclins D1 and C (*CCND1, CCNC*) and CDK inhibitors p21^CIP1/WAF1^ (*CDKN1A*), p27^KIP1^ (*CDKN1B*) and p19 (*CDKN2D*) [116,119]. The induction of p27^KIP1^ expression takes place at the promoter/transcriptional level and posttranslationally by the inhibition of its degradation [120,121,122]. These effects hamper retinoblastoma (Rb) protein phosphorylation and thus the activation of the E2F family of transcription factors, which trigger a series of target genes that are critical to entering the cell cycle from the quiescent state. In addition, an Rb-independent G_1_ arrest has been described that is probably a consequence of the repression of the *MYC* oncogene [123]. Thus, 1,25-(OH)_2_D_3_ represses *MYC* expression via direct [124] or indirect transcriptional inhibition by antagonism of the Wnt/β-catenin pathway [125,126], the induction of cystatin D [127] or the MYC antagonist MAD/MXD1 [128], by repressing long non-coding *(lnc)RNA CCAT2* [129] or by promoting MYC protein degradation [130] in several carcinoma cell types.

In some systems (colon and gastric cancer cells), 1,25-(OH)_2_D_3_ downregulates other proliferative genes such as *FOS*, *JUN*, *JUNB,* and *JUND* proto-oncogenes, *G0S2* (G_0_/G_1_ switch 2), and *CD44*, while it upregulates *GADD45A* (growth arrest and DNA damage 45a), *MEG3* (Maternally expressed gene 3, a lncRNA) and *NAT2* (N-acetyltransferase 2) [131,132,133,134]. Additionally, 1,25-(OH)_2_D_3_ induces antiproliferative genes such as *CEBPA* (CCAAT-enhancer-binding protein-α) and *IGFBP3* (insulin-like growth factor binding protein-3) in breast, prostate, or colon carcinoma cells, respectively [131,135,136]. IGFBP3 mediates the induction of p21^CIP1/WAF1^ by 1,25-(OH)_2_D_3_ in prostate carcinoma cells [136], and microRNA *miR-145* the repression of *CDK2*, *CDK6*, *CCNA2,* and *E2F3* genes and the antiproliferative effect of 1,25-(OH)_2_D_3_ in gastric cancer cells [137]. In breast carcinoma and anaplastic thyroid cancer cells, 1,25-(OH)_2_D_3_ causes G_2_/M phase arrest probably as a consequence of the downregulation of CDK2 activity due to the E2F blockade by non-phosphorylated Rb protein [138]. Vitamin D analogues also inhibit proliferation through induction of G_1_ phase arrest of some hematological cancer cells (lymphoma, myeloma, B-cell acute lymphoblastic leukemia and acute myeloid leukemia) [139].

Indirect mechanisms. 1,25-(OH)_2_D_3_ interferes with several mitogen signaling pathways in a context-dependent fashion. Thus, 1,25-(OH)_2_D_3_ decreases the expression of epidermal growth factor receptor (EGFR) and promotes its ligand-induced internalization in colon carcinoma cells [140,141]. Additionally, it diminishes EGFR signaling through the induction of E-cadherin and the repression of SPROUTY-2 and the renin-angiotensin system [125,142,143,144]. 1,25-(OH)_2_D_3_ and certain analogues interfere with the insulin-like growth factor (IGF)-I/II pathway by inhibiting IGF-II secretion and increasing IGFBP3 and IGFBP6 levels, and by inducing type II IGF receptor (IGFR-II), which accelerates IGF-II degradation and downregulates this pathway [145,146]. In oral squamous cell carcinoma cells, the 1,25-(OH)_2_D_3_ analogue Eldecalcitol antagonizes the mitogenic action of fibroblast growth factor (FGF)1/2 by repressing nuclear factor *kappa* B (NF-*k*B) and inducing *miR6887-5p*, which targets 3′UTR mRNA of heparin-binding protein 17/FGF-binding protein-1 (HBp17/FGFBP-1), a FGF2 chaperone [147,148]. In addition, 1,25-(OH)_2_D_3_ inhibits the mitogenic action of platelet-derived growth factor (PDGF)-BB in prostate cancer cells by downregulating PDGF receptor β [149]. The effect of 1,25-(OH)_2_D_3_ on hepatocyte growth factor (HGF) signaling is cell-type dependent. It is inhibitory in hepatocellular cells by reducing the expression of c-Met, the tyrosine kinase HGF receptor [150] and in promyelocytic leukemia cells by downregulating HGF RNA [151], but activating in some non-tumoral cell types [152].

1,25-(OH)_2_D_3_ also diminishes the proliferation of breast cancer cells by inhibiting estrogen synthesis and signaling through estrogen receptor (ER)α [153] and by downregulating RAS expression and the phosphorylation of its downstream effectors MEK and ERK1/2 [154]. The inhibition of pituitary transcription factor (Pit)-1 is another antiproliferative effect of 1,25-(OH)_2_D_3_ in breast cancer cells. Pit-1 expression is higher in tumors than in normal breast. It regulates growth hormone (GH) and prolactin (PRL) secretion and leads to increased cell proliferation, invasiveness, and metastasis [155]. 1,25-(OH)_2_D_3_ reduces Pit-1 expression and the increase in cell proliferation either directly or indirectly through GH and/or PRL [156].

Another indirect mechanism of the antiproliferative effect of 1,25-(OH)_2_D_3_ is the regulation of miRs. Thus, *miR-22* is induced by 1,25-(OH)_2_D_3_ and contributes to its antiproliferative effect on colon carcinoma cells 1,25-(OH)_2_D_3_ [157] and has antitumor effects in other carcinomas. Additionally, a recent study indicates that *miR-1278* sensitizes cells to 1,25-(OH)_2_D_3_ by suppressing the expression of CYP24A1 [158].

Transforming growth factor (TGF)-β is a strong inhibitor of epithelial cell proliferation in normal cells and at early steps in the tumorigenic process. 1,25-(OH)_2_D_3_ activates latent TGF-β and induces the expression of type I TGF-β receptor, which sensitizes breast and colon carcinoma cells to the growth inhibitory action of TGF-β [159,160]. Of note, TGF-β signaling is blocked in around 30% of colon cancers due to mutation of the genes encoding TGF-β receptor type II, SMAD2, or SMAD4. In contrast, TGF-β promotes at late stages epithelial-to-mesenchymal transition (EMT), migration, invasion, immunosuppression, and metastasis. As discussed in the following sections, these protumorigenic effects of TGF-β on tumor and stromal cells later in carcinogenesis are counteracted by 1,25(OH)_2_D_3_.

Concordantly with the association between low vitamin D status and poorer overall survival and progression-free survival in myeloid and lymphoid malignancies [161], in several types of leukemic cells, 1,25-(OH)_2_D_3_ regulates essential pathways for survival and proliferation such as TLR, STAT1/3 or PI3K/AKT that are induced by immune cell–cell or cytokine activation [162,163].

### 4.2. Sensitization to Apoptosis, Combined Action with Chemotherapy and Radiotherapy

Obviously, 1,25-(OH)_2_D_3_ per se does not induce apoptosis or any other type of cell death. However, it controls the expression of genes involved in apoptosis in cell systems in a way that is compatible with sensitization to the induction of apoptosis by other agents. Thus, in colon, prostate, and breast carcinoma cells, 1,25-(OH)_2_D_3_ upregulates several pro-apoptotic proteins (BAX, BAK, BAG, BAD, G0S2) and suppresses survival and anti-apoptotic proteins (thymidylate synthase, survivin, BCL-2, BCL-XL). In this way, it favors the release of cytochrome C from mitochondria and the activation of caspases 3 and 9 that lead to apoptosis promoted by a variety of signals [116,117]. Moreover, 1,25-(OH)_2_D_3_ induces apoptosis in ovarian carcinoma cells by caspase 9 activation [164] and by downregulation of telomerase reverse transcriptase (hTERT) via the induction of *miR-498* [165,166]. Intriguingly, while the aforementioned effects seem to be independent of the *TP53* gene, a study has proposed that mutant p53 protein interacts physically with VDR in breast cancer cells, converting the ligand into an anti-apoptotic agent by mechanisms that remain unclear [167].

In addition, 1,25-(OH)_2_D_3_ and metformin have additive/synergistic antiproliferative and proapoptotic effects in colon carcinoma and other types of cells, which are modulated but not hampered by *TP53* status [168]. Moreover, in an in vitro model developed to evaluate the crosstalk between tumor-associated macrophages and colon carcinoma cells, 1,25-(OH)_2_D_3_ restored the sensitivity of these cells to TRAIL-induced apoptosis by interfering with the release of interleukin (IL)-1β by macrophages [169]. Interestingly, the *TP53* mutation and suppression of *miR-17~92* polycistron are highly toxic in non-small lung cancer cell lines due to the upregulation of VDR signaling [170].

Based on these data, many completed and ongoing studies investigate the antitumor action of the combination of 1,25-(OH)_2_D_3_ and a variety of chemotherapeutic agents (5-fluorouracil, gemcitabine, paclitaxel, imatinib, and cisplatin, among others), inhibitors (of EGFR, HER2, HER4, JAK1/2 tyrosine kinases, estrogen or aromatase) and apoptosis inducers (dexamethasone, trichostatin A and 5-aza-2′-deoxycytidine, among others) in cells and animal models of several types of cancers see [116,119] and references therein. The definitive results of these studies are expected to constitute the foundation for clinical trials.

### 4.3. Regulation of Autophagy

Autophagy is a process of elimination of cytoplasmic waste materials and dysfunctional organelles that serves as a cytoprotective mechanism but that, when excessive, leads to cell death. Vitamin D activates autophagy in many organs in healthy conditions to preserve homeostasis. It can also induce autophagy as protection against cell damage caused by intracellular microbial infection, oxidative stress, inflammation, aging, and cancer [171].

In cancer, VDR ligands trigger autophagic death by inducing crucial genes in several cancer cell types. Thus, 1,25-(OH)_2_D_3_ and its analogues de-repress the key autophagic MAP1LC3B (LC3B) gene and activate 5′-AMP-activated protein kinase (AMPK) via increased cytosolic Ca^2+^ and activation of Ca^2+^/calmodulin-dependent protein kinase β in breast carcinoma cells [172]. In Kaposi’s sarcoma cells [173] and myeloid leukemia cells [174], vitamin D compounds inhibit PI3K/AKT/mTOR signaling and activate Beclin-1-dependent autophagy. 1,25-(OH)_2_D_3_ also induces autophagy through the mTOR pathway in Pfeiffer diffuse large B lymphoma cells [175] and is mediated by activation of DNA damage-inducible transcript 4 (DDIT4), in cutaneous squamous cell carcinoma cells [176]. In addition, a recent study has shown that 1,25-(OH)_2_D_3_ promotes autophagy in acute myeloid leukemia cells by inhibiting miR-17-5p-induced Beclin-1 overexpression [177].

Moreover, 1,25-(OH)_2_D_3_ or EB1089 increase radiation efficiency via promotion of autophagic cell death in a VDR- and p53-dependent fashion in non-small cell lung cancer and breast cancer cells [178,179,180,181]. Additionally, synergy between 1,25-(OH)_2_D_3_ and temozolomide in tumor reduction and prolonged survival time has been reported in rat-cultured glioblastoma cells and in an orthotopic xenograft model [182].

### 4.4. Induction of Cell Differentiation, Inhibition of Epithelial-to-Mesenchymal Transition

Cell differentiation is usually, but not necessarily, linked to an arrest in proliferation, and both processes put a brake on tumorigenesis. Carcinoma is the most frequent type of solid cancer. Carcinomas originate from the transformation of epithelial cells in a process that involves the early loss of two key features of their differentiated phenotype: apical-basal polarity and adhesiveness (cell–cell and cell–extracellular matrix, ECM). Loss of epithelial differentiation results from the acquisition of a cellular program called epithelial-mesenchymal transition (EMT), which implies changes in gene expression, triggered by a group of transcription factors (EMT-TFs: mainly SNAIL1, SNAIL2, ZEB1, ZEB2 and TWIST1). EMT provides tumor cells with features of malignancy such as migratory capacity, stemness and diminished apoptosis that facilitate invasion and metastasis and possibly cause resistance to cytotoxic chemotherapy and radiotherapy, and to immunotherapy [183]. The EMT process is activated by a variety of agents and signals that induce or activate the EMT-TFs, such as TGF-β, Wnt, Notch, and ligands of several receptors with tyrosine kinase activity and cytokine receptors.

1,25-(OH)_2_D_3_ has a prodifferentiation effect on several types of carcinoma cells either by direct upregulation of epithelial genes and/or the repression of key EMT-TFs, as shown in [184,185]. In breast cancer cells, 1,25-(OH)_2_D_3_ promotes the formation of focal adhesion contacts, structures of binding to the ECM, by increasing the expression of several integrins, paxillin and focal adhesion kinase. Additionally, 1,25-(OH)_2_D_3_ reduces the expression of the mesenchymal marker N-cadherin and the myoepithelial proteins P-cadherin, integrins α_6_ and β_4,_ and α-smooth muscle actin, which are associated with more aggressive and lethal forms of human breast cancer [186]. In colon carcinoma cells, 1,25-(OH)_2_D_3_ upregulates an array of intercellular adhesion molecules that are constituents of adherens junctions and tight junctions, including E-cadherin, occludin, claudin-2 and -12, and ZO-1 and -2 [125,131]. As mentioned by JoEllen Welsh in an excellent recent review [187], breast cancer heterogeneity is reflected in available model systems of this disease, including human breast cancer cell lines. These differ in the expression of VDR and other hormone receptors and in their global gene expression profile and phenotype. Consequently, results vary widely in laboratory studies of 1,25-(OH)_2_D_3_ and other VDR ligands, which show a heterogeneous, usually multilevel protective action that affects a variety of pathways (ERBB2/NEU-ERK-AKT, WNT/β-catenin, JAK-STAT, NF-κB, ERα). These studies have rendered only a few genes that are commonly regulated: *CYP24A1*, *CLMN*, *EFTD1* and *SERPINB1*.

Remarkably, the induction of E-cadherin by 1,25-(OH)_2_D_3_ in colon carcinoma cells has been reproduced in tumor cell lines derived from breast, prostate, non-small cell lung, and squamous cell carcinomas, usually associated with an increase in epithelial differentiation [184]. The mechanism of E-cadherin induction by 1,25(OH)_2_D_3_ in human colon cancer cells is transcriptional indirect. It requires transient activation of the RhoA-ROCK-p38MAPK-MSK1 signaling pathway [126]. Phosphatidylinositol 5-phosphate 4-kinase type II β is also needed for E-cadherin induction by 1,25-(OH)_2_D_3_ in these cells [188]. In agreement with the transcriptional regulation, 1,25-(OH)_2_D_3_ treatment causes partial demethylation of CpG sites of *CDH1* promoter in MDA-MB-231 triple-negative breast cancer cells [189]. In addition, 1,25-(OH)_2_D_3_ induces and/or redistributes several cytokeratins, F-actin, vinculin, plectin, filamin A and paxillin that modulate the actin cytoskeleton and the intermediate filament network, changing stress fibers and the ECM binding structures (focal adhesion contacts and hemidesmosomes) [125,126]. In summary, 1,25(OH)_2_D_3_ increases cell–cell and cell-ECM adhesion.

1,25-(OH)_2_D_3_ inhibits SNAIL1 and ZEB1 expression in non-small cell lung carcinoma cells, accompanied by an increase in E-cadherin expression, vimentin downregulation, and maintenance of epithelial morphology [190]. The 1,25-(OH)_2_D_3_ analogue MART-10 inhibits EMT in breast and pancreatic cancer cells through the downregulation of SNAIL1, SNAIL2 and TWIST1 in breast cancer cells [191,192]. 1,25-(OH)_2_D_3_ causes the downregulation of SNAIL1 and SNAIL2 in colon and ovarian carcinoma cells [193,194].

In addition, 1,25-(OH)_2_D_3_ induces several modulators of the epithelial phenotype that can influence the expression of these EMT-TF. Thus, it increases by a transcriptional indirect mechanism the expression of *KDM6B*, a histone H3 lysine 27 demethylase that mediates the induction of a highly adhesive epithelial phenotype in human colon cancer cells [195]. *KDM6B* depletion upregulates SNAIL1, ZEB1, and ZEB2 and increases the expression of mesenchymal markers fibronectin and LEF-1, and claudin-7. Accordingly, *KDM6B* and *SNAI1* RNA expression correlate inversely in samples from human colon cancer patients [195]. Furthermore, 1,25-(OH)_2_D_3_ directly upregulates the expression of cystatin D, which represses SNAIL1, SNAIL2, ZEB1, and ZEB2, and induces the expression of E-cadherin and other adhesion proteins such as occludin and p120-catenin. Accordingly, cystatin D and E-cadherin protein expression directly correlate in colon cancer, and loss of cystatin D is associated with poor tumor differentiation [127]. The *SPRY2* gene encodes SPROUTY-2, a modulator of tyrosine kinase receptor signaling that is strongly repressed by 1,25(OH)_2_D_3_ in colon carcinoma cells [143]. SPROUTY-2 promotes EMT through upregulation of ZEB1 and downregulation of the epithelial splicing regulator ESRP1. Consequently, SPROUTY-2 represses genes that encode E-cadherin, claudin-7, and occludin and the important regulators of the polarized epithelial phenotype LLGL2, PATJ, and ST14 [143,196].

The induction of differentiation seems to be a less important protective mechanism of 1,25-(OH)_2_D_3_ in hematological malignancies than in solid cancers. 1,25-(OH)_2_D_3_ induces differentiation almost exclusively of acute myeloid leukemia cells [197,198,199]. Thus, 1,25(OH)_2_D_3_ increases the expression of markers of the monocyte-macrophage phenotype such as CD14 and some proteins involved in phagocytosis and adherence to substratum, including CD11b [139,200]. A number of genes and proteins have been proposed as mediators of this prodifferentiation action of 1,25-(OH)_2_D_3_, such as PI3K, *CEBPB,* and *CDKN1A* [201,202,203]. Differentiation of acute myeloid leukemia cells was also described by the combination of 1,25-(OH)_2_D_3_ with l-asparaginase [204]. Interestingly, a recent study reports that liganded VDR has a strong prodifferentiation effect in acute myeloid leukemia cells harboring mutations in *IDH* gene encoding isocitrate dehydrogenase. This is the case because the oncometabolite 2-hydroxyglutarate that is produced by mutant IDH potentiates VDR signaling in a CEBPα-dependent manner [205]. In addition, prodifferentiation effects of VDR agonists have been reported in follicular non-Hodgkin’s lymphoma cells, with increased expression of mature B-cell markers [206].

### 4.5. Antagonism of Wnt/β-Catenin Signaling Pathway

The Wnt/β-catenin signaling pathway is activated by several members of the Wnt family of secreted proteins (19 in humans) during ontogenesis and adult life, which play important roles in the development and homeostasis of many tissues and organs. The binding of these Wnt factors to plasma membrane co-receptor (Frizzled-LRP) complexes inhibits the degradation of β-catenin protein in the cytoplasm that is promoted by the products of tumor suppressor genes APC and AXIN, which leads to β-catenin accumulation and partial translocation into the cell nucleus. Nuclear β-catenin acts as a transcriptional co-activator of genes bound by the T-cell factor (TCF) family of transcriptional repressors [207]. The long list of β-catenin/TCF target genes includes some that are crucial for cell survival and proliferation (MYC, CCND1), EMT, migration/invasion, and other tumoral processes (Stanford University Wnt homepage: https://web.stanford.edu/group/nusselab/cgi-bin/wnt/) (accessed on 19 March 2022). These genes are active during ontogenesis but remain mostly silent in adult life except in some situations such as wound healing. Recent data suggest that Wnt factors only prime β-catenin signaling. This causes basal activation of the pathway that only becomes fully activated in the presence of R-spondin (RSPO)1–4. Upon binding to their membrane LGR4–6 receptors, the secreted RSPO family members inactivate two E3 ubiquitin ligases (RNF43, ZNRF3) that mediate Frizzled degradation. In this way, RSPOs extend Frizzled half-life at the cell surface and so potentiate Wnt signaling.

The Wnt/β-catenin pathway is an important player in cancer as it is aberrantly activated by mutation (APC, AXIN, CTNNB1/β-catenin, RSPO2/3, and RNF43 genes), overexpression of Wnt factors/receptors or silencing of Wnt signaling inhibitors (DICKKOPF/DKKs, SFRPs) leading to the activation or potentiation of carcinogenesis [208]. This is particularly important in colorectal cancer, as massive sequencing efforts have revealed that the mutation of at least one Wnt/β-catenin pathway gene is present in over 94% of primary tumors and metastases [209,210], while a variable proportion of other cancers (liver, breast, lung and leukemia, among others) also show abnormal pathway activation. Despite its clinical relevance, no inhibitors of the Wnt/β-catenin pathway have been approved up to now.

The first description of the antagonism of the Wnt/β-catenin pathway by 1,25-(OH)_2_D_3_ was reported in colon carcinoma cells by a double mechanism: (a) liganded VDR binds nuclear β-catenin, which hampers the formation of transcriptionally active β-catenin/TCF complexes, and (b) induction E-cadherin expression that attracts newly synthesized β-catenin protein to the plasma membrane adherens junctions. In that way, it decreases β-catenin nuclear accumulation [125]. Other mechanisms of interference of the Wnt/β-catenin signaling pathway by 1,25-(OH)_2_D_3_ have been subsequently described in colon, breast, ovarian, hepatocellular, renal, head, and neck carcinomas, and in Kaposi’s sarcoma, see [211]. These mechanisms include the increase in AXIN, TCF4 or DKK1 level, modulation of TLR7, reduction of total or nuclear β-catenin, and enhancement of LRP6 degradation [212,213,214,215,216,217]. In addition, a paracrine mechanism of Wnt/β-catenin signaling has been proposed based on interruption by 1,25-(OH)_2_D_3_ of the secretion of the Wnt stimulator IL-β by environmental macrophages [218].

### 4.6. Inhibition of Angiogenesis

1,25-(OH)_2_D_3_ inhibits cancer angiogenesis by acting at two levels: tumor cells and endothelial cells. In diverse types of carcinoma cells (colon, prostate, and breast), the anti-angiogenic action of 1,25-(OH)_2_D_3_ relies to a great extent on its ability to inhibit two major angiogenesis promoters: it suppresses the expression and activity of hypoxia-inducible factor (HIF)-1α, a key transcription factor in hypoxia-induced angiogenesis, and of vascular endothelial growth factor (VEGF)-A. Additionally, 1,25-(OH)_2_D_3_ induces the angiogenesis inhibitor thrombospondin-1 [219,220]. In colon tumor cells, modulation of the angiogenic phenotype is also mediated by the control of genes encoding inhibitors of differentiation (ID)-1/2 and by the repression of DKK4, a weak Wnt antagonist that promotes angiogenesis and invasion and is upregulated in colon tumors [219,221]. 1,25-(OH)_2_D_3_ alone and more strongly in combination with cisplatin suppresses VEGF activity in ovarian cancer cells [222]. By modulating VEGF receptor (VEGFR) 2, 1,25-(OH)_2_D_3_ or calcipotriol, it enhances the efficacy of the VEGFR inhibitor Cediranib in malignant melanoma cells [223]. Another antiangiogenic mechanism of 1,25-(OH)_2_D_3_ is the reduction of IL-8 secretion by prostate cancer cells through the inhibition of NF-κB [224]. Intriguingly, variable and sometimes opposite effects of 1,25-(OH)_2_D_3_ on angiogenesis have been reported, as in a xenograft breast cancer model, where it inhibits TSP-1 and increases VEGF expression [225]. Likewise, 1,25-(OH)_2_D_3_ induces VEGF synthesis and action in some non-tumoral cell systems, see [152].

1,25-(OH)_2_D_3_ also has inhibitory effects on tumor-derived endothelial cells. It reduces their proliferation and sprouting in vitro and diminishes the blood vessel density in xenograft tumors in breast, squamous cell carcinoma, bladder and prostate cancer models [226,227,228,229,230].

### 4.7. Inhibition of Cancer Cell Migration, Invasion and Metastasis

1,25-(OH)_2_D_3_ inhibits the migratory and invasive phenotype of cancer cells as a result of its effects on the cytoskeleton and adhesive properties and on the expression of proteases, protease inhibitors and ECM proteins. To a variable extent, these effects are linked to inhibition of EMT and the TGF-β and Wnt/β-catenin signaling pathways.

As mentioned above, in carcinoma cells, 1,25-(OH)_2_D_3_ induces E-cadherin and other proteins of adhesion structures and modulates actin and intermediate filament networks, which results in increased cell–cell and cell–ECM adhesion [125,186,194,217,231,232,233]. By promoting intercellular adhesion via upregulation of E-cadherin, 1,25-(OH)_2_D_3_ suppresses prostate cancer cell rolling and adhesion to microvascular endothelial cells, which is a step in extravasation that precedes metastasis [234]. In addition, vitamin D deficiency increases breast cancer metastasis to the lung by enhancing EMT and the CXCL12/CXCR4 chemokine axis [235].

1,25-(OH)_2_D_3_ reduces breast, renal, and prostate carcinoma cell migration and invasion by downregulating the expression and/or activity of N-cadherin, the ECM components tenascin C and periostin, several integrins and metalloproteases (MMP-1, -2, and -9) and serine proteases (plasminogen activator), while it upregulates protease inhibitors and the pro-adhesive actin cytoskeleton adaptor protein PDLIM2 [236,237,238,239,240]. In triple-negative breast cancer cells, 1,25-(OH)_2_D_3_ decreases hyaluronic acid synthesis [241], and inhibits bladder cancer cell migration partially via the induction of miR-101-3p [242]. In pancreatic adenocarcinoma cells, 1,25-(OH)_2_D_3_ ameliorates the pro-invasive action of tumor necrosis factor (TNF)-α by decreasing the expression of miR-221 and increasing that of the tissue inhibitor of metalloproteinase (TIMP)-3 [243].

### 4.8. Stromal Effects: Cancer-Associated Fibroblasts

Today, the critical role of stroma in the carcinogenic process is clear. Fibroblasts are the main cellular component of tumor stroma (Cancer-Associated Fibroblasts, CAF). This is a heterogeneous cell population of multiple origins (tissue-resident fibroblasts, myeloid precursors, pericytes and adipocytes, among others) and features that is acquired via the change to an “activation phenotype”. It is thought to promote cancer invasion, angiogenesis and metastasis; inhibit the immune response; and reduce intratumoral delivery and the activity of chemotherapeutic agents [244,245]. However, the protective effects of CAF have also been described in some systems, and reprogramming their phenotype is accepted as a more advisable strategy than their elimination [246,247]. Early studies showed that VDR agonists have antifibrotic and antitumoral effects by antagonizing TGF-β in the intestine, liver, and pancreas [248,249,250,251,252].

1,25-(OH)_2_D_3_ regulated over one hundred genes in human CAF isolated from tumor biopsies of five breast cancer patients [253]. The induced gene signature reflects an antiproliferative and anti-inflammatory effect of 1,25(OH)_2_D_3_. Importantly, 1,25-(OH)_2_D_3_ inhibits the protumoral action of human colon CAF by reprograming them to a less activated phenotype. Thus, 1,25(OH)_2_D_3_ reduces the capacity of CAF to alter the ECM and their ability to promote the migration of colon carcinoma cells [254]. 1,25-(OH)_2_D_3_ regulates over one thousand genes in colon CAF that are involved in cell adhesion, differentiation and migration, tissue remodeling, blood vessel development, and the inflammatory response. Remarkably, 1,25(OH)_2_D_3_ imposes a gene signature that correlates with a better prognosis for colon cancer patients [254]. Curiously, in contrast to the antagonism reported in colon carcinoma cells, 1,25-(OH)_2_D_3_ and Wnt3A have an additive, partially overlapping effect in colon fibroblasts [255,256]. In line with the results in colon CAF, 1,25(OH)_2_D_3_ decreases the amount of miR-10a-5p found in the exosomes secreted by human pancreatic CAF, which attenuates the promigratory and pro-invasive effects that these CAF exert on pancreatic carcinoma cells [257]. Of note, a recent study reported that calcipotriol promotes an antitumorigenic phenotype of pancreatic CAF by reducing the release of prostaglandin (PG) E_2_, IL-6, periostin, and other factors. However, it reduces T-cell-mediated immunity [258]. Clearly, the action of VDR agonists on fibroblasts associated with distinct human cancers is a highly interesting, open line of research.

### 4.9. Effects on Cancer Stem Cells

Cancer stem cells (CSC) are supposedly a small population of cells present in tumors that are responsible for tumor initiation, growth, malignization, metastasis, and resistance to therapies. They originate from the mutational and epigenetic alteration of normal stem cells that maintain the homeostasis of tissues in adult life and behave as a source of new functional differentiated cells following injuries or in aging. The characterization and study of CSC present two unresolved problems: (a) the lack of confirmed universal or even tissue-specific markers, and (b) the existence of cell plasticity in tumors that implies differentiation/dedifferentiation processes during tumorigenesis and thus the lack of a stable stem phenotype but, instead, interconversion of stem and non-stem cells.

At present, there are two systems to study CSC: organoid cultures generated by CSC present in patient-derived tumor biopsies and subcultures of established, immortal tumor cell lines enriched in populations of cells expressing putative CSC markers and/or selected by their capacity to grow in suspension. Clearly, fresh, primary organoids are a more valuable system. They are three-dimensional (3D), self-organized multicellular structures generated by normal stem cells or CSC (that allow matched normal and tumor organoids to be obtained from a patient) that grow embedded in an ECM covered by a complex, tissue-specific, usually serum-free medium [259,260]. Organoids recapitulate some of the features of a particular organ or tumor of origin and are quite stable genetically, and thus are considered a better system to study cancer processes than 2D cell lines grown for decades on plastic dishes [261]. 1,25(OH)_2_D_3_ profoundly and differentially regulates the gene expression profile of colon cancer patient-derived normal and tumor organoid cultures. 1,25(OH)_2_D_3_ induced stemness-related genes (*LGR5*, *SMOC2*, *LRIG1*, and others) in normal but not tumor organoids [262]. In both normal and tumor organoids, 1,25(OH)_2_D_3_ reduced cell proliferation and the expression of proliferation and tumorigenesis genes that affected only a few Wnt/β-catenin target genes (*MYC, DKK4*). Importantly, 1,25(OH)_2_D_3_ induced some features of epithelial differentiation in tumor organoids cultured in proliferation medium, such as microvilli, adhesion structures, partial chromatin condensation, and increased cytoplasmic organelles. These effects were also observed in rectal tumor organoids [263].

Concordantly, 1,25(OH)_2_D_3_-regulated genes were involved in cell proliferation, differentiation, adhesion, and migration in another study using patient-derived colon organoids [264]. Moreover, MDL-811, an allosteric activator of the sirtuin (SIRT)6 deacetylase, reduced cell proliferation in colon carcinoma cell lines and patient-derived organoids and has a synergistic antitumoral effect in combination with vitamin D in *Apc*^min/+^ mice [265]. However, conflicting data have been found in normal, nontumoral organoids: whereas 1,25-(OH)_2_D_3_ increased the stemness genes and the undifferentiated associated cell phenotype in organoids from healthy colon and rectum tissues of a dozen individuals [262,263], it enhanced the differentiation of organoids established from a benign region of a radical prostatectomy from a single patient [266].

A series of studies have examined the action of VDR agonists on putative breast cancer stem or progenitor cells identified by some markers (CD44^hi^/CD24^low^ and/or ADH1^+^) that can grow as floating, nonadherent spheres (mammospheres). In these systems, 1,25(OH)_2_D_3_ or the BXL1024 analogue reduced the population of putative CSC and the formation of mammospheres and the expression of pluripotency markers (OCT4, KL-4), Notch ligands and target genes, and genes involved in proliferation, EMT, invasion, metastasis, and chemoresistance 32,467,291 [267,268,269].

Organoids formed by cells isolated from patient-derived xenografts (not obtained directly from human biopsies but on injection and growth in mice) that acquired resistance in vitro to Trastuzumab-emtansine (T-DM1; composed of the humanized monoclonal anti-HER2 antibody Trastuzumab covalently linked to the microtubule-inhibitory agent DMI) constitute an intermediate system to the two discussed above. In this system, two vitamin D analogues (UVB1 and EM1) reduce the formation and growth of organoids [270].

### 4.10. Effects on the Immune System

1,25-(OH)_2_D_3_ is an important modulator of the immune system, as reflected by the expression of VDR by almost all types of immune cells [271,272,273]. 1,25-(OH)_2_D_3_ is an enhancer of innate immune reactions against infections and tumor cells by activating the responsive cells (macrophages, natural killer (NK) cells, and neutrophils). Conversely, and in line with its accepted anti-inflammatory action (that may contribute to the inhibition of cancers associated with chronic inflammation), 1,25-(OH)_2_D_3_ is commonly presented as a repressor of the adaptive immune reactions by deactivating antigen-presenting cells (induction of tolerogenic dendritic cells) and CD4^+^ type-1 helper T (Th1) response (production of interferon-γ, IL-1, IL-6, IL-12...), and by promoting the suppressive Th2 and Treg responses (production of IL-10, IL-4, IL-5, IL-13...) [273,274]. Moreover, in macrophages, 1,25-(OH)_2_D_3_ has been proposed to promote a switch from the pro-inflammatory M1 phenotype (producing IL-1β, IL-6, TNF-α, RANKL, COX) towards the anti-inflammatory protumoral M2 phenotype and to reduce the T-cell stimulatory capacity of macrophages [275,276]. This is somehow counterintuitive as it would represent a potential protumoral effect that cannot be easily attributed to a conserved evolutionary agent such as vitamin D. Some other studies discussed below have introduced putative explanations.

Since naïve T-cells express VDR at a very low level that increases only after activation of the T-cell receptor [277], the role of 1,25-(OH)_2_D_3_ may conceivably be related to the late downregulation of the activated adaptive response. This view agrees with the usual description of repressive 1,25-(OH)_2_D_3_ action in experimental settings following overstimulation of the cells, and it may constitute a safety mechanism to prevent undesirable long-lasting immune activation, potentially leading to inflammation or autoimmunity [278,279]. Concordant with this idea and the anticancer action of 1,25-(OH)_2_D_3_, a series of studies have revealed antitumor effects at the level of several types of immune cells.

Interestingly, a study in mice orthotopically implanted with breast tumors has revealed that vitamin D decreases tumor growth and increases the amount of tumor-infiltrating cytolytic CD8+ T-cells, a usual marker of antitumor response. This effect is lost in high-fat diet conditions [280]. Moreover, in pancreatic cancer, 1,25-(OH)_2_D_3_ inhibits the T-cell suppressive function of myeloid-derived suppressor cells [281].

An important mechanism of 1,25-(OH)_2_D_3_ is the inhibition of the NF-κB pathway. In turn, this causes the downregulation of multiple cytokines and their effects [282]. 1,25-(OH)_2_D_3_ inhibits NF–κB at different levels: by inactivating the p65 subunit of the NF-κB complex and upregulating the inhibitor subunit IκB. In addition, 1,25-(OH)_2_D_3_ inhibits the PG-endoperoxide synthase (PTGS-2, also known as COX-2) [283,284,285]. 1,25(OH)_2_D_3_ reduces the protumorigenic effect of PG E_2_ in prostate cancer cells by inhibiting COX-2 and so decreasing the levels of PG E_2_ and two PG receptors (EP2 and FP) [286]. Importantly, vitamin D and calcium favorably modulate the balance of expression of COX-2 and 15-hydroxyPG dehydrogenase, its physiological antagonist, in the normal-appearing colorectal mucosa of patients with colorectal adenoma [287]. Vitamin D enhances the tumoricidal activity of NK cells and macrophages [288,289]. 1,25-(OH)_2_D_3_ probably has a dual effect of stimulating the differentiation from monocytes to macrophages and their cell killing activity, including antibody-dependent cell cytotoxicity (ADCC). It may later balance these effects by promoting the M1 to M2 phenotypic switch ([279] and references therein). In addition, 1,25-(OH)_2_D_3_ enhances the susceptibility of hematological and solid cancer cells to NK cell cytotoxicity through downregulation of *miR-302c* and *miR-520c* [289].

The potentiation of ADCC of macrophages and NK cells may be a relevant antitumor action of 1,25-(OH)_2_D_3_ in clinical cases, particularly in patients treated with antibodies, of which the major mechanism of action is ADCC. Thus, several studies have shown that vitamin D deficiency impairs the macrophage and/or NK cell-mediated cytotoxicity of Rituximab (anti-CD20) in diffuse large B-cell, follicular, and Burkitt lymphoma patients [288,290,291], and of Cetuximab (anti-EGFR) in colon cancer cell lines [292]. In addition, some evidence of benefit has been observed in breast cancer patients treated with Trastuzumab (anti-HER2) and in melanoma patients treated with Bevacizumab (anti-VEGF) [290,293].

Agents that target programmed death (PD)-1 or its ligand PD-L1 immune checkpoint inhibitors (ICI) have attracted great attention in cancer therapy. Interestingly, 1,25-(OH)_2_D_3_ upregulates PD-L1 in human (but not mouse)-cultured epithelial and immune cells [294], while vitamin D treatment increases PD-1 expression in CD24^+^CD25^+int^ T-cells in Crohn’s disease patients [295] and PD-L1 in epithelial and immune cells in melanoma patients [296]. These data suggest the possibility of combined treatments with VDR agonists and these ICIs, and perhaps others in development.

In conclusion, it is conceivable that 1,25-(OH)_2_D_3_ works as a general homeostatic regulator of the immune system, ensuring an appropriate global defense against challenges like tumors and infections.

### 4.11. Animal Models

Many studies on animal diet, chemical, genetic, and xenograft models (mainly for colon and breast cancer) have shown the antitumor actions of vitamin D compounds. This in vivo action is difficult to dissect and probably results from a variable combination of mechanisms in the distinct systems that were assayed, including the inhibition of tumor cell growth, EMT, invasiveness, angiogenesis, and metastasis. Importantly, as occurs in cultured cancer cells, vitamin D antitumor action is mostly independent of *TP53* gene status [119,187].

### 4.12. Systemic Effects: Detoxification and Microbiome

#### 4.12.1. Detoxification

The elimination of xenobiotics or the detoxification process involves chemical modification (phase I reactions: oxidation, hydrolysis, etc.) and subsequent conjugations to water-soluble molecules (phase II reactions) carried out by a large number of enzymes. 1,25-(OH)_2_D_3_ regulates some of these enzymes in the intestine and liver [297]. This may have a positive effect on the prevention of tumorigenesis and perhaps another more controversial impact on the inactivation of chemotherapeutic drugs [298].

1,25-(OH)_2_D_3_ induces CYP3A4, a major human drug-metabolizing enzyme, SULT2A, a phase II sulfotransferase, and members of the multidrug resistance-associated protein (MRP) family in colon carcinoma cells [299,300]. CYP3A4, SULT2A1, and MRP3 are involved in the elimination of lithocholic acid (LCA), a secondary bile acid LCA that induces DNA damage and inhibits DNA repair enzymes in colonic cells. Accordingly, LCA promotes colon cancer in experimental animals, and high levels of LCA have been found in colon cancer patients [301,302]. Interestingly, LCA binds weakly and activates VDR, and so it activates its own degradation [303]. Another example is enhancement by 1,25(OH)_2_D_3_ of the benzo[a]pyrene metabolism via CYP1A1 in macrophages [304].

#### 4.12.2. Microbiome

Alteration of the intestinal microbiome (dysbiosis) is connected to colon cancer and possibly other neoplasias [305]. Many experimental studies in mice have shown that vitamin D deficiency promotes gut permeability, colon mucosa bacterial infiltration, and translocation of intestinal pathogens. These effects lead to changes in immune cell populations and gut inflammation, and cancer—an overall condition that is improved after vitamin D supplementation [306,307]. As bacteria lack VDR, the effect of vitamin D is mediated by the host. Importantly, genome-wide association analysis of the gut microbiome in two large cohorts of individuals identified VDR as a factor that influences the gut microbiota [308]. A conditioned medium from probiotic lactic acid bacteria showed increased expression of VDR and of its target *CAMP* gene encoding cathelicidin in cultured colon carcinoma cells and organoids. It protected against the inflammatory response induced by TNF-α [309]. The protective action against dysbiosis and the intestinal tumorigenesis of liganded VDR have been proposed to be at least partially mediated by the inhibition of the JAK/STAT pathway [310].

### 4.13. Discussion of Mechanistic Studies

The vast array of effects that 1,25-(OH)_2_D_3_ has in a wide variety of experimental systems of a high number of cancer types agrees with a selected evolutionary role in protection against tumoral processes. The underlying mechanisms include the control of tumor cell survival (autophagy, apoptosis) and phenotype (differentiation), and the inhibition of their proliferation, invasiveness, and metastasis; attenuation of the proliferation and phenotypic features of some CSC; modulation of the physiology of diverse non-tumoral stromal cells (fibroblasts, endothelial cells); and the regulation of several types of immune cells and responses. Table 11 summarizes the references corresponding to key studies focused on the most relevant topics of the anticancer action of vitamin D.

**Figure 3:**
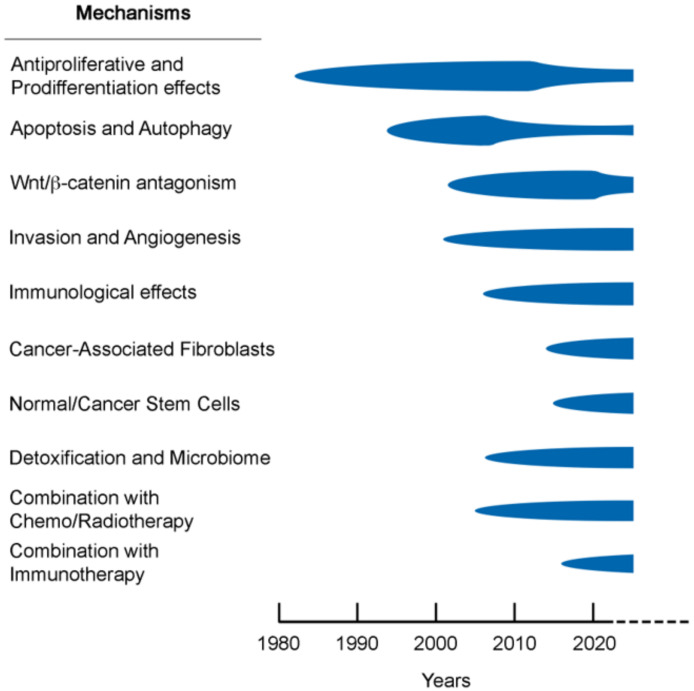
Time flow-chart of studies on the anticancer mechanisms of vitamin D compounds with some key references that are discussed in the text.

Together, these effects reflect a multilevel anticancer action of vitamin D. Therefore, an appropriate vitamin D status of the organism should be maintained to minimize the risk and severe consequences of many neoplasias. Further supporting this, the toxicity of vitamin D supplementation is limited, acceptable, and clearly lower than that of current anticancer drugs and therapies. We are not aware of any other natural or synthetic compound that has such an array of antitumor activities combined with low toxicity. Doubtless, the available experimental results meet Koch’s postulate for biological causality regarding the existence of a global mechanism of action behind the association between vitamin D deficiency and high incidence and, especially, the mortality of several major cancer types found in observational and epidemiological studies. Hopefully, the further development of current and possibly, novel studies on the wide range of mechanisms of VDR agonists in a variety of biological systems will allow us to elucidate the anticancer action of vitamin D (Figure 3).

## 5. Outlook

On the basis of this review of ecological and observational studies, it seems that an efficient way to strengthen the links between vitamin D and cancer is to conduct more CC studies of cancer incidence. Such studies would measure 25(OH)D concentration, C-reactive protein, and other relevant factors, as well as obtain the history of UVB exposure, vitamin D supplementation, and dietary sources of vitamin D. The next step is to then find appropriate controls using, perhaps, the propensity score analysis, as done in a study of breast cancer survival with respect to de novo vitamin D supplementation [311]. In addition, care should be taken to investigate the effect of vitamin D supplementation and 25(OH)D concentration on cancer risk for various subgroups based on such factors as age, BMI, diet, ethnicity, geographical location, etc.

Future laboratory research on the anticancer action of vitamin D is desirable to develop a deeper understanding of the individual response to treatment with VDR agonists. To this end, *omics* studies using genomic, epigenomic, transcriptomic, proteomic, and metabolomic approaches must be integrated to understand and foresee personal susceptibility/sensitivity to each compound, which has been defined as “the personal vitamin D response index” [312]. Clearly, the characterization of biomarkers of compound activity and patient response in different cancer types will be important. Since 1,25-(OH)_2_D_3_ regulates the same pathways but distinct genes of them in mice and humans [313], studies should preferentially be carried out in human systems. Among them, it seems that primary cell cultures and organoids should be used instead of classical, long-term established cell lines.

Given the increasingly important role attributed to the stroma in tumorigenesis, the effects of vitamin D compounds on CAF, endothelial cells, and specific types of immune cells require attention. Likewise, the association of chronic inflammation with several types of cancer and the pro-inflammatory action of adipocytes suggest the interest in studying the effects of vitamin D in this context.

Another open field for research is combination therapies. Up until now, experimental studies have focused on the combination of VDR agonists and chemotherapeutic agents, sometimes with radiotherapy. Obviously, this should be continued and extended to the exponentially growing field of cancer immunotherapies.

## Figures and Tables

**Table 1:** Characteristics of large single-country ecological studies of cancer incidence or mortality rates with respect to solar UVB doses.

Country(ies)	Solar UVB Index	Latitude (°N)	Incidence or Mortality; Years of Data	No. of Cases	Confounding Factors	Ref.
U.S.	Surface UVB, July 1992, TOMS	25–45	Mortality, 1950–1994	9.5 million, 1970–1994	None	[13]
Japan	Annual hours of solar radiation	30–45	Mortality, 2000	180,000	Fat intake for colon, rectum, and prostate; salt intake for stomach cancer	[21]
U.S. (white pop.)	Surface UVB, July 1992	25–45	Mortality, 1950–1994	9.5 million, 1970–1994	Alcohol consumption, Hispanic heritage, lung cancer (index for smoking), poverty, urban/rural residence	[14]
U.S.	300–320 nm, TOMS, north vs. south	25–45	Incidence, 1998–2002; mortality, 1993–2002	Incidence, 3.4 million; mortality, 3.5 million	Age, air quality, alcohol, exercise, income, outdoor occupation, poverty, smoking, urban/rural residence	[22]
Japan	Global solar radiation	30–45	Mortality, 1998–2002	~900,000	Dietary factors, smoking, socioeconomic conditions	[23]
China	TOMS, 305 nm	22–50	Incidence, 1998–2002; mortality, 1990–1992		Urban/rural residence	[18]
Russia	Latitude	43–69	Incidence, mortality, 2008	incidence, ~250,000; deaths, ~140,000	None	[24]
Nordic countries	Lip cancer less lung cancer incidence	55–70	Incidence, 1961–2005	2.8 million	Lung cancer	[25]

Pop., population; TOMS, NASA’s Total Ozone Mapping Spectrometer satellite instrument.

**Table 2:** Ecological studies of cancer incidence rates with respect to indices of solar UVB doses.

Incidence [29] (×1000)	Cancer	USA [22]	China [18]	Russia [24]	Nordic [25]
219.4	Lung		–M, FNS, –R, –U		M, FNS
194.3	Breast	F	–F, –R, –U		M, F
192.3	Prostate	M		–M	MNS
147.0	Colorectal		M, F, R		
106.1	Colon	M, F			M, F
71.0	Bladder, urinary	M, F	–M, –F, –R, –U		M, F
68.7	Melanoma	–M, –F		M + F	M
66.0	Non-Hodgkin lymphoma	M, F			NS
57.8	Kidney	M, F		M + F	M, FNS
44.8	Leukemia	M, F	MNS, FNS, R, –U		
42.5	Pancreas	M, F		M + F	M, FNS
42.2	Uterus, corpus	F			FNS
40.9	Rectum	M, F			M, FNS
37.2	Thyroid	MNS, F			
35.7	Oral cavity and pharynx	–M, –F			
23.1	Oral				M
22.6	Myeloma	M, F		M + F	
22.6	Liver		–M, –F, –R, –U		M, FNS
22.1	Brain				M
21.6	Ovary	FNS			
21.1	Stomach (gastric)	M, F	M, F, R, –U	M + F	M?, FNS
16.5	Esophagus	M	M, F, R, –U	M + F	MNS
12.6	Pharynx		–M, –F, –R, –U	–(M + F)	
12.3	Larynx				M
11.3	Cervix	–F	F, R,–U		
9.8	Gallbladder	F			M
9.8	Biliary, other	M, F		M + F	
8.5	Hodgkin lymphoma	M, F			
8.4	Testis				NS
6.2	Small intestine	M, F			M
5.9	Skin, other	–M, –F		–(M + F)	–M
5.3	Anus, etc.	–M, –F			
3.6	Vulva	F			

F, female; FNS, female nonsignificant; M, male; MNS, male nonsignificant; R, rural residence; U, urban residence, –, direct correlation; ?, uncertain.

**Table 3:** Ecological studies of cancer mortality rates with respect to indices of solar UVB doses.

Mortality [29] (×1000)	Cancer	Japan [23]	USA [14]	USA [22]	China [18]	Russia [24]
159.4	Lung	M, F			M, F, R, U	
69.1	Colorectal	M			M, F, R	
49.9	Colon		M, F	M, F		M + F
40.6	Breast	FNS	M, F	F	F, R	–(M + F)
35.2	Pancreas	M, F	M, FNS	M, F		M + F
27.4	Prostate	MNS	MNS	M		M
21.9	Leukemia			M, F	MNS, FNS	
19.5	Non-Hodgkin lymphoma		M, F	M, F		
19.2	Rectum		M, F	M, F		M + F
18.2	Liver	M		–M, –F	M, F, R	
14.6	Ovary		F	F		F
14.5	Esophagus	M	M, F	M	M, F, R	M + F
14.3	Bladder, urinary		M, F	M, F	M, F, R	M + F
13.9	Kidney		M, F	M, F		M + F
12.9	Brain			–M, –F		
10.6	Myeloma			M, F		M + F
10.6	Stomach (gastric)	M, FNS	M, F	M, F	M, F, U	M + F
8.7	Melanoma			–M, –F		M + F
7.8	Uterus, corpus		F	F		
7.6	Oral cavity and pharynx			–M, –F		
5.4	Oral		MNS, FNS			
4.1	Cervix		F	–F	–F, –R, –U	
3.7	Larynx		M, F?	MNS, FNS		M + F
3.4	Gallbladder	MNS, F	M, F	M, F		
3.4	Biliary, other			M. F		
2.9	Skin, other			–M, –F		–(M + F)
2.2	Pharynx				–M. –F, –R, –U	
1.6	Thyroid			MNS, F		
1.5	Bone and joint			–M, –F		
1.3	Hodgkin lymphoma		M, F	M, F		
1.1	Small intestine			MNS, F		
0.9	Vulva			F		F
0.7	Anus, etc.			–M, –F		M + F

F, female; FNS, female nonsignificant; M, male; MNS, male nonsignificant; R, rural residence; U, urban residence, –, direct correlation; ?, uncertain.

**Table 4:** Data related to Figure 2 in McCullough and colleagues [47].

Study	Follow-Up (Years)	RR	Ref.
**Men**			
ATBC2	12.5	1.17	[48]
PHS	9.50	1.06	[49]
CLUE II	3.20	0.99	[50]
HPFS	6.30	0.99	[51]
JANUS	5.10	0.93	[52]
EPIC	3.60	0.86	[53]
MEC	1.50	0.86	[54]
CPS-II	3.20	0.83	[55]
JPHC	5.10	0.83	[56]
CARET	4.90	0.82	[57]
PLCO	5.40	0.81	[58]
ABCT1	3.50	0.77	[59]
**Women**			
ORDET	10.8	1.03	[60]
JPHC	5.10	0.94	[56]
JANUS	5.10	0.90	[52]
BGS	2.30	0.90	[61]
CLUE-II	9.00	0.87	[50]
WHI	3.20	0.87	[62]
NHS	9.60	0.84	[51]
CPS-II	3.20	0.77	[55]
WHS	8.00	0.77	[63]
EPIC	3.60	0.73	[53]
NYUWHS	12.3	0.72	[64]
PLCO	5.40	0.67	[58]
MEC	1.50	0.63	[54]

ATBC, Alpha-Tocopherol, Beta-Carotene Cancer Prevention Study; BGS, Breakthrough Generations Study; CARET, Carotene and Retinol Efficacy Trial; CLUE II, Cancer Prevention Study II Nutrition Cohort; CPS-II, Cancer Prevention Study II; EPIC, European Prospective Investigation into Cancer and Nutrition; HPFS, Health Professionals Follow-up Study; JANUS, JANUS Serum Bank, Norway; JPHC, Japan Public Health Center-Based Prospective Study; MEC, multiethnic cohort study’; NYUWHS; New York University, Women’s Health Study; ORDET, Hormones and Diet in the Etiology of Breast Cancer Risk; PHS, Physicians’ Health Study; PLCO, Prostate, Lung, Colorectal and Ovarian Cancer Screening Trial; RR, relative risk; WHI, Women’s Health Initiative.

**Table 5:** Meta-analyses of observational studies of incidence risk of individual cancer sites related to serum 25(OH)D concentration.

Cancer Site	*N* Studies, Cases, Controls	Type of Study	Follow-Up (Years)	RR (95% CI), High vs. Low	Ref.
All	8, —, —	Prospective, incidence	5–28	0.86 (0.73–1.02)	[70]
All	17, —, —	Prospective, mortality	5–28	0.81 (0.71–0.93)	[70]
Bladder	5, 1251, 1332	CC and NCC, incidence	0 (4), 12, 13	0.70 (0.56–0.88)	[71]
Bladder	2, 2264, 2258	Cohort, incidence	14, 28	0.80 (0.67–0.94)	[71]
Breast	44, 29,095, 53,060	CC and NCC, incidence		0.57 (0.48–0.66)	[68]
Breast	6, 2257, —	Cohort, incidence		1.17 (0.92–1.48)	[68]
Colorectal	11, —, —	1 CC, 9 NCC, 1 meta-analysis, incidence	0–20	0.60 (0.53–0.68)	[72]
Colorectal	6, 1252, —	Cohort, incidence	8–20	0.80 (0.66–0.97)	[72]
Colorectal	15, 6691, —	NCC, incidence		0.67 (0.59–0.76)	[73]
Head and neck	5, —, —	Cohort, incidence	7, 15	0.68 (0.59–0.78)	[74]
Liver	8, 992, —	Cohort, incidence	6–28	0.78 (0.63–0.95)	[75]
Liver	6, 776, —	Cohort, incidence	(0.75), 16–22	0.53 (0.41–0.68)	[76]
Lung	8, 1386, —	Cohort, incidence	7–26	0.72 (0.61–0.85)	[77]
Lung	9, —, —	7 Cohort, 2 CC, incidence		0.84 (0.74–0.95)	[78]
Lung	3, —, —	1 Cohort, 2 CC, mortality		0.76 (0.61–0.94)	[78]
Lung	12, —, —	7 Cohort, 5 CC		1.05 (0.95–1.16)	[79]
Ovarian	8, —, —	CC, cohort, NCC		0.86 (0.56–1.33)	[80]
Pancreatic	5, 1068, —	2 Cohort, 3 NCC, incidence	6.5–21	1.02 (0.66–1.57)	[81]
Pancreatic	5, 2003, —	Cohort, mortality	6.5–21	0.81 (0.68–0.96)	[81]
Prostate	19, 12,786	16 NCC, 3 cohort, incidence		1.15 (1.06–1.24)	[82]
Renal	5, —, —	4 Cohort (+1 CC, 3.5% weighting), incidence	(0), 7–22	0.76 (0.64–0.89)	[83]
Renal	1, —, —	CC, incidence	0	0.30 (0.13–0.72)	[83]
Thyroid	6, 387, 457	CC, incidence	0	Deficiency, 1.30 (1.00–1.69), *p* = 0.05	[84]

95% CI, 95% confidence interval; CC, case–control study; NCC, nested case–control study; parentheses for follow-up years indicate numbers for a very small percentage of the total; RR, relative risk; —, no data.

**Table 6:** Meta-analyses of observational studies of the risk of incidence of individual cancer sites related to vitamin D intake.

Cancer Site	*N* Studies	Type of Study	RR (95% CI), High vs. Low Vitamin D Intake	Ref.
Breast	17	8 CC, 9 cohorts	0.97 (0.92–1.07), per 400 IU/d	[68]
Colorectal	12	CC	0.75 (0.67–0.81)	[72]
Colorectal	6	Cohort	0.89 (0.80–1.02)	[72]
Head and neck	3		0.75 (0.58–0.97)	[74]
Lung	6	Cohort	0.89 (0.83–0.97)	[77]
Lung	5	Cohort	0.85 (0.74–0.98)	[79]
Renal	4	CC	0.80 (0.67–0.95)	[83]
Renal	4	Cohort	0.97 (0.77–1.22)	[83]
Overall cancer death			0.84 (0.74–0.95)	[87]

CC, case–control study; NCC, nested case–control study.

**Table 7:** Estimates of odds ratio for maximum 25(OH)D concentration compared with minimum concentration for several cancers.

Cancer	Min 25(OH)D (ng/mL)	Max 25(OH)D (ng/mL)	OR (95% CI)	Ref.
All, inc	2	25	~0.6	[70]
Bladder, inc	3	30	~0.55 (0.35–0.70)	[71]
Breast, inc (Song et al.)	5	85	~0.2 (0.1–0.3)	[68]
Breast, inc	15	70	0.18 (0.04–0.62)	[69]
Colorectal, inc	4	55	~0.4 (0.3–0.5)	[73]
Colorectal, inc	10	50	~0.7 (0.4–1.0)	[88]
Liver, inc	4	30	0.35 (0.21–0.48)	[76]
Liver, inc	5	30	~0.6 (0.5–0.7)	[75]
Lung, inc	6	21	0.87 (0.76–0.97)	[89]
Lung, inc	10	24	0.80 (0.61–0.98)	[78]
Lung, mort	10	42	0.37 (0.25–0.53)	[78]
Prostate, inc	0	60	~1.3 (1.1–1.8)	[82]
Prostate, mort	4	43	~0.55 (0.2–1.1)	[90]

Inc, incidence; mort, mortality; OR, odds ratio.

**Table 8:** Characteristics of ten RCTs that investigated the effect of vitamin D supplementation on risk of cancer incidence and/or mortality rate.

Location	Mean Baseline and Achieved 25(OH)D (ng/mL), Treatment Arm	Vitamin D Dose (IU) Frequency in Treatment Arm	Duration (Years)	Mean BMI (kg/m^2^)	Original Purpose	Reference
UK		100,000/ (4 months)	5.5	24 ± 3	fracture incidence, cause of death	[93]
USA		400/day + 1 g/day Ca	7	28?	colorectal cancer incidence, mortality	[94]
Nebraska, USA	29, 38	1100/day + 1.5 g/day Ca; 1.5 g/day Ca	4	29 ± 6	fracture incidence	[95]
Australia	21, 24–48	500,000/year			falls and fractures	[96]
England, Scotland		800/day; 1 g/d Ca; 800/day + 1 g/day Ca	3			[97]
Nebraska, USA	33, 44	2000/day + 1500 mg/day Ca	4	30 ± 7	cancer	[98]
New Zealand	26, --	100,000/mo	3.3 ± 0.8	28 ± 5	disease incidence with respect to bolus dose of vitamin D	[99]
USA	30, 41	2000/day	5.3	31	cancer and cardiovascular disease risk	[85]
Australia	31 ± 10, 46 ± 12	60,000/ month	5	27?	mortality by disease	[100]

**Table 9:** Outcomes of ten RCTs that investigated the effect of vitamin D supplementation on risk of cancer incidence and/or mortality rate with respect to intention to treat.

Location	Number of Participants, Cancer Cases, Deaths, Treatment Arm	Number of Participants, Cancer Cases, Deaths, Non-Vitamin D Arm	RR, Incidence (95% CI)	RR, Mortality (95% CI)	Reference
UK	1345, 163, 63	1341, 147, 72	1.11 (0.86–1.42)	0.86 (0.61–1.20)	[93]
USA	18,176, 1634, 344	18,106, 1655, 382	0.98 (0.91–1.05)	0.89 (0.77–1.03)	[94]
Nebraska, USA	446, 13, --	733, 37, --	0.76 (0.38–1.55)		[95]
Australia	1131, 7	1125, 10	0.70 (0.27–1.82)		[96]
England, Scotland	1306, 182, 78; 1311, 189, 95	1343, 187, 73; 1332, 165, 83	1.24 (0.80–2.28)	1.26 (0.73–3.26)	[97]
Nebraska, USA	1156, 45, --	1147, 64, --	0.70 (0.47–1.02)		[98]
New Zealand	2558, 302, --	2550, 293, --	1.01 (0.81–1.25)		[99]
USA	12,927, 793, 154	12,946, 824, 187	0.96 (0.88–1.06)	0.83 (0.67–1.02)	[85]
Meta-analysis for ten incidence trials and five mortality rate trials			0.98 (0.93–1.03)	0.87 (0.79–0.96)	[86]
Australia	21,315, --, 221	10,662, --, 189		1.15 (0.96–1.39)	[100]

**Table 10:** List of epidemiological studies that had important findings in the history of solar UVB exposure and/or vitamin D and cancer.

Year	Finding	Reference
1936	Sun exposure can cause skin cancer but reduce risk of internal cancer.	[1]
1937	US Navy personnel highly exposed to sun had high skin cancer rates but low internal cancer rates.	[2]
1941	Cancer mortality rates for whites in the U.S. found inversely related to a solar radiation index while skin cancer (melanoma) mortality rates were directly related.	[3]
1980	Annual solar radiation dose inversely correlated with colon cancer mortality rate, USA, vitamin D production suggested.	[6]
1985	Dietary vitamin D and calcium inversely correlated with colorectal cancer incidence.	[7]
1989	Serum 25(OH)D concentration inversely correlated with colon cancer incidence.	[8]
1990	Annual solar radiation dose inversely correlated with breast cancer mortality rate in the U.S.	[9]
2002	Mortality rates for thirteen types of cancer are inversely correlated with solar UVB doses in the U.S., 1970–1994.	[13]
2006	A Harvard cohort study finding that incidence of several types of cancer were inversely correlated with predicted 25(OH)D concentration.	[111]
2006	An ecological study in the U.S. finding that incidence and mortality rates for many types of cancer were inversely correlated with solar UVB doses.	[22]
2007	A meta-analysis presenting a 25(OH)D concentration-colorectal cancer incidence relationship.	[46]
2007	An RCT conducted in the U.S. finding that vitamin D supplementation significantly reduced risk of all-cancer incidence rate.	[95]

**Table 11:** Vitamin D anticancer mechanisms in experimental model systems. List of key representative references.

Mechanism	Cancer Type Model	References
Inhibition of cell proliferation	Breast, prostate, colon, ovarian, gastric thyroid, hepatocellular, leukemias, lymphomas	[111,119,120,121,122,123,124,125,126,127,128,129,130,131,132,133,134,135,136,137,138,139,140,141,142,143,144,145,146,147,148,149,150,152,153,154,155,156,158,159,161,162]
Induction of differentiation	Leukemia, colon, breast	[112,124,125,126,138,176,185,187,196,197,198,199,200,201,202,203,204,205]
EMT inhibition	Colon, ovarian, breast, pancreas	[126,142,189,190,191,192,193,194,195]
Sensitization of autophagy	Colon, prostate, breast, ovarian, lung	[115,116,118,163,164,165,168,169]
Induction of autophagy	Breast, Kaposi’s sarcoma, lymphoma, cutaneous squamous cell carcinoma, leukemia	[171,172,173,174,175,176,177,178,179,180,181]
Wnt/β-catenin antagonism	Colon, breast, ovarian, hepatocellular, renal, head and neck, Kaposi’s sarcoma	[124,210,211,212,213,214,215,216,217]
Invasion, angiogenesis, metastasis	Colon, prostate, breast, ovarian, renal, pancreas	[193,194,195,196,197,198,199,200,201,202,203,204,205,206,207,208,209,210,211,212,213,214,215,216,218,219,220,221,222,223,230,231,232,233,234,235,236,237,238,239,240,241,242]
Cancer-associated fibroblasts	Breast, colon, pancreas, liver	[248,250,252,253,254,255,256,257]
Normal/cancer stem cells	Breast, colon, pancreas, liver	[261,262,263,264,265,266,267,268,269]
Detoxification and microbiome	Colon, perhaps other cancer types	[296,297,298,299,300,301,302,303,305,306,307,308,309]
Immune system regulation	Many	[272,273,274,275,276,277,278,279,280,281,282,283,284,285,286,287,288]
Combination with immunotherapy	Lymphoma, melanoma, colon, breast	[289,290,291,292,293,294,295]
